# Er-Doped Nanostructured BaTiO_3_ for NIR to Visible Upconversion

**DOI:** 10.3390/ma11101950

**Published:** 2018-10-12

**Authors:** Ariel Meneses-Franco, Marcelo Campos-Vallette, Sergio Octavio Vásquez, Eduardo A. Soto-Bustamante

**Affiliations:** 1 NSC Nanosono SA, R&D Corporation, Hakidma 7, Yokneam industrial Park 2069200, Israel; akahlil@gmail.com; 2 Department of Chemistry, Faculty of Science, University of Chile, Las Palmeras 3425, Ñuñoa, Santiago 780003, Chile; facien05@uchile.cl; 3 Department of Materials Science, Faculty of Physical and Mathematics Sciences, University of Chile, Beauchef 850, Santiago 837048, Chile; svasqueza@ing.uchile.cl; 4 Department of Organic Chemistry and Physical Chemistry, Faculty of Chemical and Pharmaceutical Sciences, University of Chile, Sergio Livingstone 1007, Independencia, Santiago 8380492, Chile

**Keywords:** upconversion, photoluminescent, nanoparticle, Barium Titanate, Erbium

## Abstract

Photoluminescent mechanisms in erbium-doped barium titanate nanoparticle systems were studied. Er^3+^ ions were introduced into the BaTiO_3_ lattice by the sol-gel method. The resulting Er^3+^ concentration was between 0% and 5%, with Ba/Ti ratios of 1.008 and 0.993. The stoichiometry of Ba and Ti concentrations in the lattice influenced the doping mechanism and placement of erbium ions in the lattice structure. Our research shows the existence of a strong correlation between Ba/Ti ratios, erbium concentration, phase structure and doping site location on the upconversion photoluminescence mechanisms. Competing upconversion emissions ^2^H_11/2_/^4^S_3/2_→^4^I_15/2_ at 523 and 548 nm respectively and other photoluminescent mechanisms as ^4^I_9/2_→^4^I_11/2_ around 4000 nm (2500 cm^−1^) were studied using Raman and emission spectroscopy. The upconversion process is predominant over other photoluminescent decay when the material presents high distortion in the surrounding activator.

## 1. Introduction

The development of nanoscience in the last decade has allowed the advancement of diverse areas [1], including biomedicine and pharmaceutical technology [2,3,4]. Polymeric systems for controlled drug release aim to obtain devices whose control is more efficient and less invasive [5,6]. Some systems have been developed including the photomechanical properties of molecules such as azo-benzenes. Due to the irradiation with light of a certain wavelength, the structure of the material is modified [7,8,9,10]. It has great advantages in vitro, but great limitations for in vivo application: it requires UV irradiation sources, which are harmful to the tissues and far from the so-called optical window of them.

Different components in tissues have different absorption values and therefore different molar extinction coefficients (ε), often used to describe their absorption of photons. The penetrability of light in tissues depends on the abundance of chromophores. Water, lipids and hemoglobin in their free and oxygenated form are the most common species. Taking this into account, it is possible to determine their optical window, which is in the span of 700 nm and 900 nm [11].

Because near infrared light (NIR) is less harmful and has better penetrability in tissues, this paper postulates that it is possible to use upconversion systems to transform light of NIR wavelengths into visible wavelengths, suitable for generating conformational transitions in azo-compounds. In this way, it would be possible to irradiate tissues to reach devices composed of polymeric azo-compounds containing specific drugs and Er-doped BaTiO_3_ nanosystems. In this way, it will be possible to release these drugs directly to the site of action of the active principle.

As a result of the existence of the so-called optical window in the span of 700 nm and 900 nm, phenomena of emission of anti-Stock light have been studied; these phenomena are produced in amorphous and crystalline materials doped with transition elements, especially lanthanides. When they are irradiated with light of a certain wavelength, they will emit light at lower wavelengths in a process that involves absorption of two or more photons, depending on the mechanism [12]. In this sense, the upconversion phenomena is one of the mechanisms responsible for the IR to visible light conversion. Considering our previous experiences with nanomaterials [13], we explore the feasibility of incorporating lanthanides into ferroelectric materials, taking special care of the physical properties of the new ceramics.

**Figure 1:**
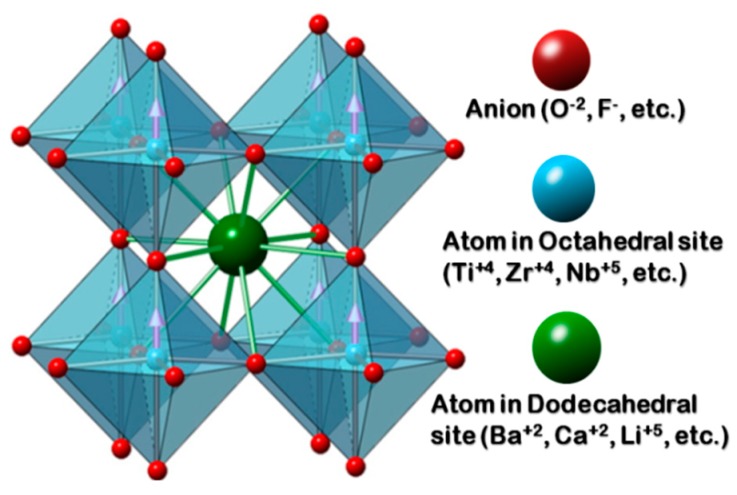
Typical scheme for a perovskite lattice structure.

Perovskites as barium titanate (BaTiO_3_, BT), with an ABO_3_ general formula, have been extensively studied due to their ferroelectric properties [14], which also involves piezoelectricity, pyroelectricity [15] and a high dielectric constant [16]. Structurally, perovskites form a crystal lattice based on oxygen or other anions in an octahedral arrangement, which generate two types of cavities: one with octahedral symmetry to accommodate small cations, generally tetra or pentavalent, and the other with dodecahedral symmetry, where cations of varying sizes, usually mono or divalent, can be hosted [17] (Figure 1). According to this, different cations may be accommodated in the crystalline lattice, either at the octahedral or dodecahedral site, depending on charge and ionic radius. These inserted ions create distortions of the original lattice which modify the material properties [18].

Divalent cations such as Sr^2+^, Ca^2+^, and even Pb^2+^ have been incorporated into the dodecahedral site of BT, resulting in significant changes in the Curie temperature (T_C_) which allow for the modification of the operating temperature of these materials. In the case of Pb^2+^, this produces an increase in T_C_ and an improvement of the dielectric constant [19]. Sr^2+^ and Ca^2+^ produce a decrease in T_C_ [20,21]. Other examples of the versatility of the perovskite structure include the incorporation of donor impurities at the octahedral site, such as Zr^4+^ or Nb^5+^. Depending on the concentration of these ions, both the T_C_ and dielectric constants may be modified. This will influence the semiconducting or insulator behaviors with a subsequent improvement in dielectric breakdown strength in the positive temperature coefficient resistivity (PTCR effect) [22,23]. Moreover, transition metals such as Co^3+^, Ni^2+^, Cr^3+^, which prefer to stay at the octahedral site acting as acceptors, reduce the mobility of ferroelectric domains, thus improving their usefulness as capacitors [24,25,26].

Other interesting families of dopants for perovskites are constituted by Y^3+^ and the lanthanide series (Ln^3+^). Changes in the dielectric properties by the incorporation of these elements in the lattice [27] are produced. They also change the luminescent properties of these materials through Stokes and anti-Stokes fluorescence emission, for example, by upconversion. This generally consists of a luminescent process, wherein the non-radiative interaction between an ion pair may generate luminescent emission of higher frequency than the excitation.

Possible upconversion mechanisms are Excited State Absorption (ESA) and Energy Transfer Upconversion (ETU) [28,29].

In the 4*f*→4*f* transitions, the influence of the surroundings close to the optically excited activator is small but significant. Upconversion occurrence, as well as other photoluminescent processes present in the lanthanide ions, are dependent on probabilistic selection rules. Therefore, they compete with each other, producing in many cases a quenching phenomenon [12]. BT possesses interesting spectroscopic characteristics, such as a low energy phonon [30], as well as high chemical stability, which makes it an excellent candidate for hosting luminescent ions.

The incorporation of Ln^3+^ ions into the BT structure may occur either at the octahedral sites or at the dodecahedral sites. Its insertion will be influenced by the ion’s effective charge and the charge-structure compensation performed between the matrix and the lanthanide ionic radius. This last characteristic is the most critical factor in incorporating rare earth ions into ceramics [31]. Some authors have proposed a statistical theoretical study to determine the affinity of a lanthanide for one or the other crystalline site. However, in the case of Ln^3+^ atoms with ionic radii and intermediate charge ranging between 1.35 Å for Ba^2+^ and 0.68 Å for Ti^4+^, it is not theoretically possible to predict this trend due to the proximity of the calculated energies for the proposed implantation processes [32]. One of the most interesting cases, due to their applications, is for Er^3+^ [33]. At least four different incorporation mechanisms have been proposed which thermodynamically favor the incorporation at both octahedral and dodecahedral sites. Therefore, the synthesis conditions, will determine the insertion site and the compensation mechanisms that may be involved [34].

Several methods have been developed for BT nanoparticles (BTNp) synthesis and the study of doping processes requires a synthetic pathway which ensures the homogeneity of the material components as well as the purity of the obtained product. The hydrothermal or solvothermal methods [35] may produce fairly uniform nanoparticle sizes which can be dispersible in organic media, but with many impurities. Pyrolysis of micro droplets [36] produces highly pure materials, but with a significant size dispersion and expensive equipment is required.

Another method is the synthesis of a sol-gel precursor prepared from organic acids and subsequent thermal treatment [37]. This method completely eliminates water and also provides a free ionic product, with a small number of carbonates adsorbed at the nanoparticle surface. It is based on the formation of a macromolecular organometallic complex, which will be decomposed at high annealing temperatures. In this method, after removal of solvents, the remaining organics decompose and subsequently form complex oxides. The final product is obtained when it reaches the amorphization temperature, which is around 850 °C for BT [38]. Then, the system is cooled down at room temperature, yielding high purity nanopowders with tetragonal symmetry [13,17]. With this synthetic method, it is possible to adjust certain conditions such as the O_2_ partial pressure, annealing temperature, Er^3+^ concentration or Ba/Ti ratio (R), among others which will finally produce changes in the material properties [39].

The structural distortions induced by the foreign ion implantations in the surrounding environment generate modifications which are reflected in the lattice spectroscopic properties, as well as in certain physicochemical phenomena. Therefore, it is essential to consider the vibrational changes experienced by the material [40,41,42]. In this work, we study the structural changes generated by the implantation of Er^3+^ into BTNp in concentrations varying between 0% and 5%, with a Ba/Ti ratio (R) of two.

## 2. Materials and Methods

**Figure 2:**
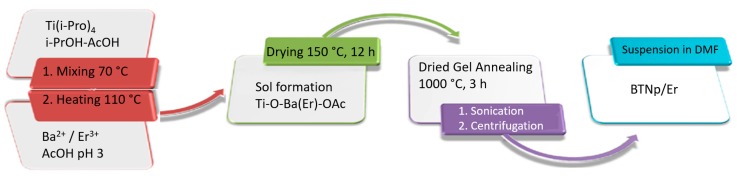
Process outline for the synthesis of erbium/barium titanate nanoparticles (Er/BTNp). DMF = dimethylformamide.

All precursors and solvents are of commercial grade from Merck KGA (Darmstadt, Germany) or Aldrich (Saint Louis, Missouri, MO, USA). The preparation of BTNp was as follows (Figure 2): in a two-necked flask placed on a heating mantle, 25 mL of dry 2-propanol and 15 mL (49.5 mmol) of titanium tetraisopropoxide were mixed. Then, 34 mL of glacial acetic acid was added, with constant stirring between 50–60 °C for 30 min. Simultaneously, 9.5 g (50 mmol) of barium hydroxide monohydrate is dissolved in hot glacial acetic acid and the required amount of Er(NO_3_)_3_·5H_2_O is added, thus defining the final Er^3+^ concentration in BT.

The temperature of the solution with titanium tetraisopropoxide is raised to 70 °C with constant stirring and the acetic Ba/Er containing solution is added dropwise. Once mixed, the temperature is raised to 110 °C and as a result, a clear and homogeneous sol is obtained which is dried at 150 °C for 12 h, yielding small yellowish crystals of a dehydrated gel. This is transferred to porcelain crucibles and annealed for 3 h at 1000 °C to obtain a white nanopowder. The synthesis was repeated ten times, adjusting the erbium concentration and the barium hydroxide/titanium tetraisopropoxide proportions (0.998 R < 1 and 1.008 R > 1), to study the effect of the R in the structural characteristics of the modified BTNp. Finally, the crude product of BT is suspended in dimethylformamide (DMF), submitted to ultrasound and centrifuged at 4500 rpm to obtain a milky BTNp suspension.

For size distribution, the samples were studied by Transmission Electron Microscopy (TEM) using a Zeiss microscope, model EM-109 in copper grids as support, which were covered with FORMVAR^®^ (SPI Supplies, West Chester, PA, USA) for aqueous samples or carbon for organic samples, operating at 80 kV. The TEM images were analyzed with the AxioVision Rel. 4.8 (Zeiss, Jena, Germany) image processing software [13].

A Differential Thermal Analyzer (DTA) (FP90 DTA (Mettler Toledo, Madrid, Spain) was used to investigate the thermal behavior of the samples using aluminum crucibles. The DTA was calibrated using different standards: benzophenone (ME18870) 47.9 ± 0.2 °C; benzoic acid (ME18555) 122.3 ± 0.2 °C and caffeine (ME18872) 236.0 ± 0.3 °C. As a control, we used indium (ME119442) 156.6 °C at a ramp rate of 4 °C/min (±0.1 °C).

X-ray diffraction measurements of the nanopowders were performed in a D5000 diffractometer (SIEMENS-BRUKER, Münich-Karlsruhe, Germany), using CuKα radiation (1.5418 Å). The room temperature-controlled samples contained D_K_ concentric plate rings with radii of 5 cm.

The IR spectra of nanopowders were obtained in a FT-IR Bruker IFS28 spectrophotometer (Bruker, Karlsruhe, Germany) operated by OPUS software in transmission mode; the samples were dispersed in KBr spectroscopy grade powder.

The Raman spectral analysis was performed at room temperature in backscatter using a Renishaw Micro-Raman System 2000 equipped with the 633 nm and 785 nm laser sources, a Leica microscope and an electronically cooled CCD detector (Charge Coupled Device). Spectra were scanned under selected instrumental conditions. The spectra were obtained using a 50× objective. The laser power was set at 1–10 mW on the sample with a resolution of 4 cm^−1^, collecting 1–5 scans of 10–30 s each.

## 3. Results

**Figure 3:**
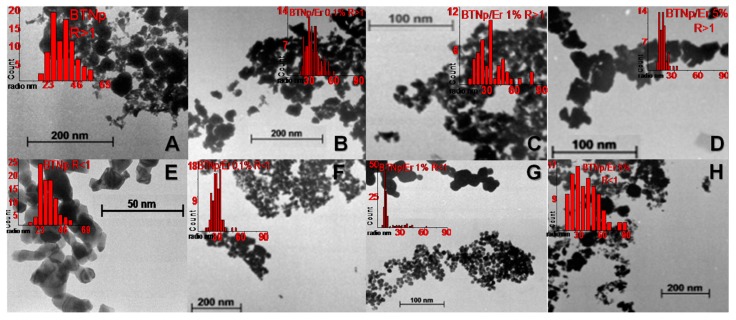
Transmission electron microscopy (TEM) images and histograms for the size distribution of BTNp at different Ba/Ti ratios (R) and app. % *w*/*w* erbium content: Top R > 1, A (0%); B (0,1%); C (1%) and D (5%); Bottom R < 1 E (0%); F (0,1%); G (1%) and H (5%).

We studied the influence of R in its crystalline structure before modifying the BTNp crystalline lattice. From the analysis of the TEM images, we can observe polygonal shaped nanoparticles with high dispersion size in both samples. For samples with R > 1 (Figure 3a–d), there is a certain population of small particles of irregular shape which are difficult to characterize using imaging and which are not affected by the Er^3+^ concentration. They probably correspond to small fragments produced by the ultrasound treatment. There always exists a different population of more compact and regular ovoid-like particles, being of more abundance for the R < 1 samples. Foremost in the R < 1 case, TEM images (Figure 3e–h) revealed the fact that by increasing the Er^3+^ concentration in the samples, we obtain larger nanoparticles with a higher size dispersion.

**Figure 4:**
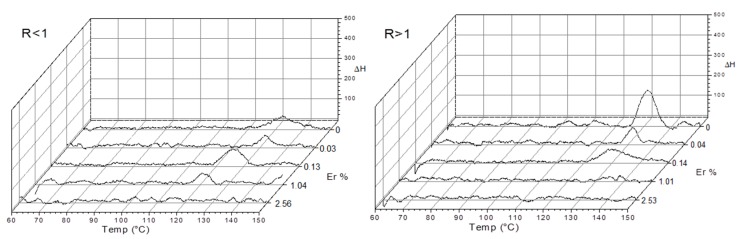
Er/BTNp thermograms for the two ratios of Ba/Ti with different concentrations of Er^3+^.

Barium titanate (BaTiO_3_) is a ferroelectric oxide that undergoes a transition from a ferroelectric tetragonal phase to a paraelectric cubic phase upon heating above 130 °C [43]. DTA measurements show a dissimilar behavior for both Ba/Ti ratios at this characteristic transition from tetragonal to cubic phase (Figure 4). For R > 1, the transition suffers a sustained decrease in intensity at the same temperature while the Er concentration increases, and disappears over 0.14%. Samples with R < 1 conserved this tetragonal-to-cubic phase transition which gradually disappears above 1%, with a temperature displacement from 130 °C to 120 °C. This means that below 120 °C in the temperature range studies, for R < 1, erbium can be located in a tetragonal lattice at concentrations ten times higher compared with R > 1.

**Figure 5:**
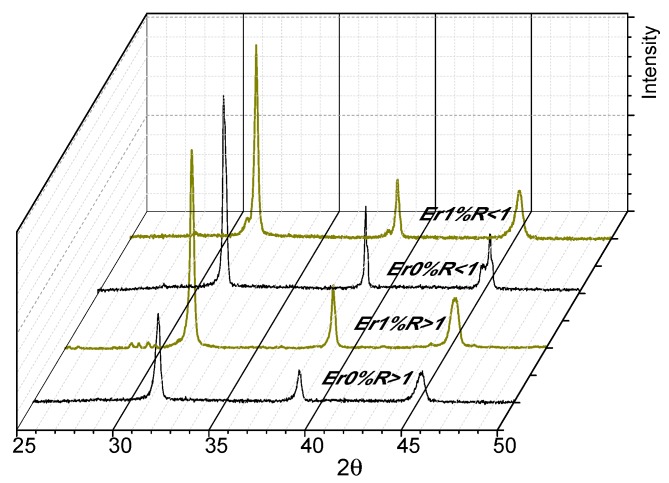
XRD for R > 1 and R < 1 of BTNp and Er/BTNp 1%.

The results of X-ray diffraction show the tendency to tetragonality due to the characteristic decoupling of the 200-002 system close to 2θ = 45° in R > 1 undoped (see Figure 5, Er 0% R > 1). A peak broadening can also be seen which, according to the Scherrer equation, corresponds to a reduction of particle size which also agrees with the presence of a great number of fine particles for R > 1 (Figure 3a). Such particles were not included in the statistical analysis. This is supported by calorimetric data (Figure 4) which show an increase in the tetragonal (t-BT) to cubic (c-BT) signal for the phase transition of BT near 125 °C. X-ray diffractograms of different samples (Figure 5) also show differences for both Ba/Ti ratios.

The pattern for R > 1 is not significantly distorted, although small signals between 28° and 30° in 2θ do appear (Figure 5, Er 1% R > 1) which are not assignable to the original lattice. This is the result of a lattice distortion produced by the doping process with erbium ions which have a higher atomic radius, thus displacing elements in the crystalline network of the BT samples.

**Figure 6:**
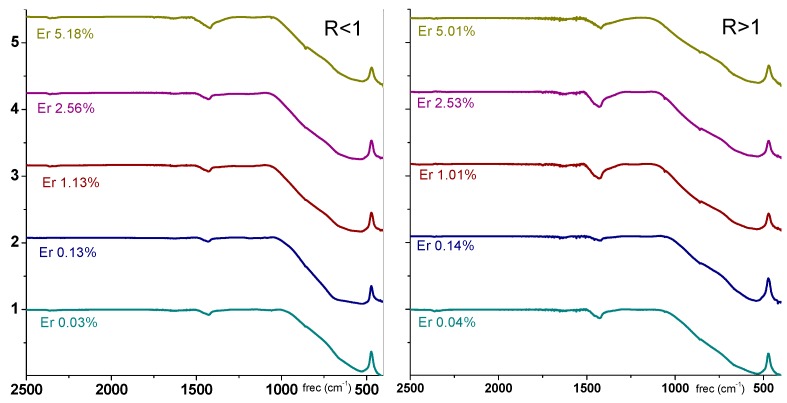
FT-IR spectra of samples top R > 1, bottom < 1 at different Er^3+^ percentages.

The FT-IR spectrum also shows evidence of barium carbonates (around 1400 cm^−1^) or barium and titanium oxides (shoulder in the 1000–900 cm^−1^ region) [42] (Figure 6); these components are not incorporated in the BT lattice. The analysis of FT-IR spectroscopy for R > 1 (Figure 6, right) for an increase in Er^3+^ concentration shows that the intensity of the 1425 cm^−1^ and 900 cm^−1^ bands decreases; they are now more resolved. For R < 1 (Figure 6, left) the signals are weaker and narrow. Then, for samples of low concentrations of erbium ions, we conclude that Er^3+^ preferentially displaces barium for R > 1 samples. On the contrary, Er^3+^ prefers to displace titanium for R < 1, partly changing the stoichiometry of BT.

The IR spectra of potential contaminants, available on request (obtained following a similar synthesis and not shown here), make it plausible to associate them to BaO, BaCO_3_ and TiO_2_ in some proportion. Webler et al. studied the influence of the presence of barium carbonate (BaCO_3_) phase on the luminescence properties of barium titanate nanocrystal (BaTiO_3_) powders using Fourier transform infrared spectroscopy and X-ray powder diffraction [44]. They identified the presence of impurities related to BaCO_3_. The presence of BaCO_3_ at trace levels reduces the infrared-to-visible upconverted luminescence efficiency of the produced nanopowders.

**Figure 7:**
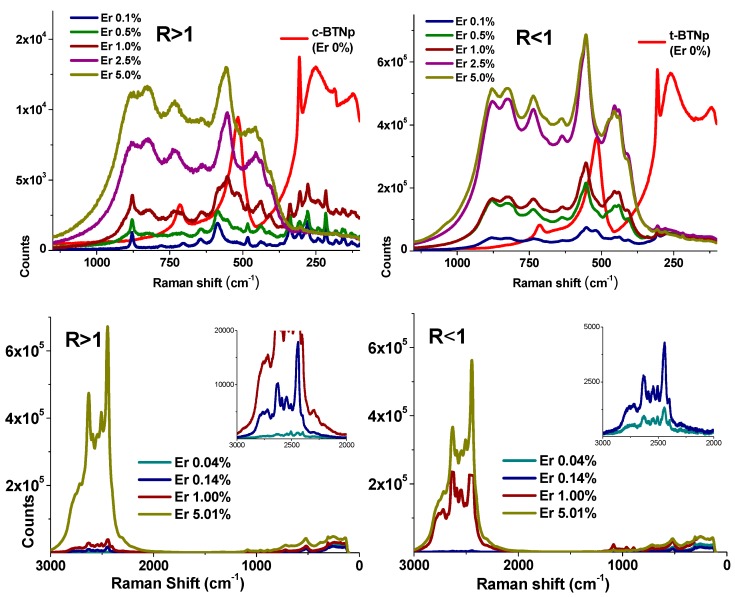
Raman spectra at 633 nm (**top**) and 785 nm (**bottom**), of samples R > 1 and R < 1 at different Er^3+^ concentrations.

The influence of R on the Raman spectral profile of the undoped BT structure is evident from Figure 7 (red curves). The most evident spectral change for the t-BT structure using the 633 nm laser line is the drastic intensity decrease of the signal around 182 cm^−1^ (Figure 7 top, red curves). Such spectral behavior could be related to feeble interactions between atoms in the horizontal planes when passing from c-BT to t-BT, in agreement with conclusions from thermal modifications studies [45]. The Raman spectra of doped BT materials in R < 1 samples, irradiated with 633 nm, along with the undoped material, display spectral changes by erbium increasing (Figure 7, right). For R > 1 at low erbium concentrations, the BT spectrum disappears completely and is replaced by the spectrum of BaTi_2_O_5_ [41] (Figure 7, left). These sets of signals gradually form a unique and identical spectrum for 1 < R < 1 at concentrations larger than 2.5%. Since Er^3+^ displays an absorption band near 800 nm, we found resonant Raman information by using the 785 nm laser line, thus making it possible to infer the influence of the host lattice on the erbium site symmetry (Figure 7 bottom).

## 4. Discussion

Lazarevic et al. prepared BaTiO_3_ ceramic powders; the formation of phase and its crystal structure of BaTiO_3_ were studied by X-ray diffraction analysis and Raman spectroscopy. Most of the Raman-active modes for tetragonal BaTiO_3_ (P4 mm) 4E(TO + LO) + 3A1(TO + LO) + B1(TO + LO) were observed and discussed [46]. Here LO is the Longitudinal optical and TO the transverse optical splitting on internal modes in the Raman spectra. Da-Yong Lu et al. found enhanced and abnormal Raman signals from (Ba_(1 − x)_Er_x_)Ti_(1 − x)_O_3_ (x = 0.01) (BET) ceramic inferring the site occupations of Er^3+^ ions. The spectral intensity of BET is approximately one hundred times higher than that of undoped BaTiO_3_. They also concluded that BaTiO_3_ doped with Ti-site Er^3+^ shows the common Raman phonon modes of the tetragonal BaTiO_3_ [47]. Kumar et al. reported a study on a series of lanthanum-doped barium stannate-titanate (LBTS) ceramics towards correlating their microstructure with ferroelectric properties. In order to study the impact of La addition on relaxor behavior, the co-doping of Sn and La in barium titanate was investigated using Raman spectroscopy [48].

In general, inorganic materials have vibrational signals involving the metallic moieties below 1000 cm^−1^ and particularly phononic modes in the 200–10 cm^−1^ range. Spectral regions over 1000 cm^−1^ are usually not scanned; however, outstanding and unexpected vibrational Raman signals could be active due to different photoluminescent mechanisms. In the case of lanthanides for instance, various 4f electronic states with different spin multiplicity could be involved in the transition mechanisms of upconversion processes. This process is represented by Raman signals far from the excitation laser line and equivalent to a new involved electronic transition. For instance, in the present research, for the new bands around 2500 cm^−1^ plus 12,739 cm^−1^ (equivalent to 785 nm, the excitation line used), the result is 15,259 cm^−1^ equivalent to 656 nm. The energy increase is evident.

It is known that more than one mechanism exists for the inclusion of erbium in the BT [32], which produces a perturbation of the original matrix in order to compensate for defects in charge and size produced when a different ion is accommodated in perovskites. In our case, when R < 1, t-BT is preferably obtained and the addition of small amounts of erbium distorts the structure to c-BT (Figure 4, Er 1% R < 1). In this case, this indicates that erbium ions have a very good solubility in the crystal, changing the structure to a cubic lattice.

From Raman analysis (Figure 7), for both R ratios the spectra are very similar in the 100–2000 cm^−1^ spectral region; however, for the 2000–3000 cm^−1^ range, there are severe spectral changes: (a) new signals appear for both cases, (b) only in R > 1, a drastic intensity increase is verified from 1% to 5% of erbium and (c) for R < 1, a gradual spectral intensity increase is observed. The 2000–3000 cm^−1^ spectral profile is consistent with the photoluminescent transitions of lanthanides [49,50]. Thus, it is probable that the structural distortion imposed by the Er^3+^ concentration changes and induces the appearance of the observed photoluminescent signals at different Ba/Ti ratios.

**Figure 8:**
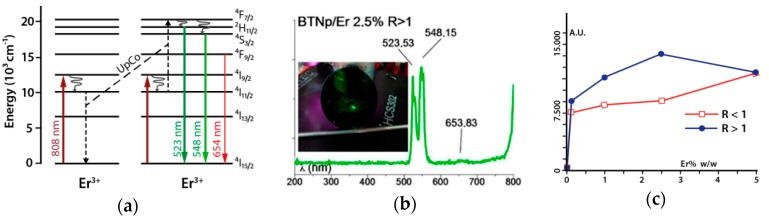
(**a**) Upconversion processes in Er^3+^ in BTNp/Er after excitation with 808 nm NIR laser radiation; (**b**) Emission spectra and visible emission of BTNp/Er 2.5% R < 1; (**c**) Relative intensity emission versus Er^3+^ concentration in both Ba/Ti ratios.

Samples of BTNp/Er were excited under 808 nm NIR laser radiation. It is well known that in Er^3+^ upconversion, processes do occur during excitation with NIR radiation at the proper wavelength. In this case, the 808 nm excitation populates the ^4^I1_9/2_ state, which undergoes a non-radiative process to the intermediate ^4^I1_1/2_ state. An upconversion to the ^4^F_7/2_ state could be triggered by the presence of more equally excited erbium cations at distances close enough to facilitate the interaction and energy transfer. Subsequent non-radiative internal relaxation populates the ^2^H_11/2_ and ^4^S_3/2_ states, from which radiative decay to the ground state causes the green fluorescence observed at 524 nm and 548 nm, respectively (Figure 8a). There is also very weak evidence of emission at 654 nm (^4^F_9/2_). All these results are in good agreement to Ghosh et al. [51], Hao et al. [33] and Huang, Han et al. [52].

Measurements of the relative emission at this wavelength for both R ratios and six different Er^3+^ concentrations show a distinctive behavior for low erbium doping. In general, for samples up to a concentration of 2.5% Er^3+^, samples with R > 1 emit a more intense green fluorescence (Figure 8c). This agree well with the fact that in such systems, there is a population of Er^3+^ ions situated in distorted sites of lower symmetry different than octahedral or tetrahedral sites. As the final intensity of the green emission depends on both the oscillator strength of the upconversion process 〈I11/24|μ→|4F7/2〉 and the subsequent oscillator strengths of the processes for decay to the ground state 〈H11/22|μ→|I15/24〉 and 〈S3/24|μ→|I15/24〉, and bearing in mind that the lower the site symmetry, the bigger the magnitude of the electric dipole transition matrix elements, the Er^3+^ ions in sites distorted by the appearance of BaTi_2_O_5_ obviously produce a much more efficient upconversion mechanism than the corresponding R < 1 samples. This behavior is still observable at 2.5% doping and R > 1 because of the shorter average distances between the few Er^3+^ ions at distorted sites that enhances the effect. For concentrations of 5%, the relative intensity is the same for both R because the lattice becomes the same for this concentration.

## 5. Conclusions

In this work, BaTiO_3_ nanoparticles doped with Er^3+^ (BTNp/Er) were synthesized by the sol-gel method. Two different Ba/Ti ratios (1.008 R > 1 and 0.993 R < 1) were studied with the purpose of understanding which sites erbium occupies in the crystalline lattice. For R > 1, inclusion of Er^3+^ at low concentration drastically disturbed the tetragonal symmetry of undoped BTNp. This matched with characteristic signals of the BaTi_2_O_5_ stoichiometry, responsible for higher distortion of the original structure. This behavior starts to change when Er^3+^ is doped over 1%, producing a common structure independently of the Ba/Ti ratio. The R is the determinant in the erbium inclusion mechanism in the BTNp crystalline lattice.

All samples exhibit upconversion green luminescence, which is also dependent on the R and erbium concentration. Excitation using a NIR laser beam of 808 nm populates the ^4^I_9/2_ state of Er^3+^ ions. As mentioned above, the most probable mechanism to explain green emission is an ETU upconversion process that involves two Er^3+^ ions in the ^4^I_11/2_ intermediate state that match the ^4^F_7/2_ state. Then, efficient green emissions at 523 nm (^2^H_11/2_) and 548 nm (^4^S_3/2_) are produced. The use of an 808 nm excitation beam avoids ESA upconversion processes, due to the fact that they should match higher energetic levels, such as ^4^F_3/2_ or even ^4^G_9/2_, and a much more intricate and less probable mechanism of multi-phonon non-radiative relaxations to the ^2^H_11/2_ and ^4^S_3/2_ emissive states.

A structural modification is inferred from the Raman data; for R > 1 at low erbium concentrations, a relevant photoluminescent behavior is inferred from the Raman spectrum; the symmetry characteristics of Er^3+^ determine a competition between both luminescent effect and upconversion. Then, it is possible to drastically modify the luminescent properties of the present material by controlling its structural parameters.

According to the above, we can conclude that the upconversion process is predominant over other photoluminescent decays when the material presents high distortion in the surrounding activator. These doped nanoparticles may be good candidates to be used in complex controlled drug release devices useful for upconverting IR light into visible light near the tissues in the study.
